# Excessive Replacement of Fish Meal by Soy Protein Concentrate Resulted in Inhibition of Growth, Nutrient Metabolism, Antioxidant Capacity, Immune Capacity, and Intestinal Development in Juvenile Largemouth Bass (*Micropterus salmoides*)

**DOI:** 10.3390/antiox13070809

**Published:** 2024-07-04

**Authors:** Hualiang Liang, Mingchun Ren, Lu Zhang, Haifeng Mi, Heng Yu, Dongyu Huang, Jiaze Gu, Tao Teng

**Affiliations:** 1 Key Laboratory of Integrated Rice-Fish Farming Ecology, Ministry of Agriculture and Rural Affairs, Freshwater Fisheries Research Center, Chinese Academy of Fishery Sciences, Wuxi 214081, China; 2 Tongwei Agricultural Development Co., Ltd., Key Laboratory of Nutrition and Healthy Culture of Aquatic Livestock and Poultry, Ministry of Agriculture and Rural Affairs, Healthy Aquaculture Key Laboratory of Sichuan Province, Chengdu 610093, China; 3 Wuxi Fisheries College, Nanjing Agricultural University, Wuxi 214081, China

**Keywords:** replacement of fish meal, soy protein concentrate, nutrition metabolism, antioxidant capacity, immune capacity

## Abstract

This study investigated the effects of replacing 0% (SPC0), 25% (SPC25), 50% (SPC50), 75% (SPC75), and 100% (SPC100) of fish meal (FM) with soy protein concentrate (SPC) on the growth, nutritional metabolism, antioxidant capacity, and inflammatory factors in juvenile largemouth bass (*Micropterus salmoides*) (17.03 ± 0.01 g). After 56 days of culturing, various growth parameters including FW, WGR, and SGR were not significantly different among SPC0, SPC25, and SPC50 groups; however, they were significantly higher than those in SPC75 and SPC100 groups. Conversely, significantly lower FCR were determined for the SPC0, SPC25, and SPC50 groups compared with that for the SPC100 group; specifically, no significant difference among SPC0, SPC25, and SPC50 groups was found. Moreover, compared with SPC75 and SPC100 groups, a significantly higher FI was observed in the SPC0 group, whereas a significantly lower SR was observed in SPC100 compared with that in SPC0 and SPC25 groups. Compared with the SPC0 group, significantly lower mRNA levels of *tor*, *rps6*, *4ebp1*, *pparγ*, and *fas* were found in SPC75 and SPC100. Additionally, the mRNA levels of *cpt* were significantly higher in SPC0, SPC25, and SPC50 groups than in SPC75 and SPC100 groups. Moreover, the mRNA levels of *scd* and *acc* remained unchanged for all the groups. Replacement of FM with SPC did not significantly affect the mRNA levels of *gk*, *pk*, and *pepck*. Compared with the SPC0 group, significantly decreased activities of CAT were observed in the SPC50, SPC75, and SPC100 groups, and significantly decreased activities of GSH-Px were observed in the SPC75 and SPC100 groups. In addition, significantly lower activity of SOD was observed in SPC100 compared with the other groups. Moreover, compared with the other groups, the SPC75 and SPC100 groups had significantly decreased and increased contents of GSH and MDA, respectively, while significantly lower mRNA levels of *nrf2*, *cat*, *sod*, and *gsh-px* were found in SPC50, SPC75, and SPC100; however, significantly higher mRNA levels of *keap1* were observed in SPC75 and SPC100 groups. Additionally, significantly higher mRNA levels of *il-8* and *nf-κb* were found in the SPC50, SPC75, and SPC100 groups compared with the SPC0 group. Conversely, significantly lower mRNA levels of *il-10* and significantly higher mRNA levels of *tnf-α* were found in the SPC75 and SPC100 groups compared with the other groups. Compared with the SPC0 group, mucosal thickness and villus height were significantly decreased in the SPC75 and SPC100 groups. Collectively, SPC replacing 50% FM did not affect its growth of juvenile largemouth bass. However, SPC replacing 50% or more FM might inhibit antioxidant capacity and immune capacity to even threaten the SR, resulting in impaired intestinal development in replacing FM level of 75% or more.

## 1. Introduction

Sustainable aquaculture ensures food security by providing high-quality protein worldwide [1]. Compared with 2015, aquatic feed production is expected to increase by 33% by 2025 [2]. Therefore, reducing the use of raw materials in feed production is crucial for ensuring the sustainable development of the aquaculture industry [2,3]. The growth and health of fish are largely affected by the availability of adequate nutrients irrespective of the farming system used [4]. Alternatively, the quality of the main protein source in the feed affects the nutritional value of fish [5]. In aquaculture, fish meal (FM) is considered a high-quality protein source due to the balanced composition of amino acids, protein content, antinutritional factors, unsaturated fatty acids, and so on [6]. However, FM is a majorly unsustainable ingredient in aquatic feed due to the overfishing of the ocean and its rising cost resulting from the continuous development of the aquaculture industry [3]. Moreover, there are limited or no prospects for increasing FM production in the future [7]. Nevertheless, inconsistency in the supply and price of FM pose considerable risks; therefore, identification, development, and utilization of FM alternatives remain a high-priority strategy for risk management in sustainable aquaculture development [8].

Soybean protein concentrate (SPC) is obtained by extracting defatted soybean flakes using water, ethanol, or methanol, and it has digestible protein and energy with better palatability compared to soybean meal; therefore, it is considered a quality protein source to replace FM [9]. In addition, most of the antinutritional factors of SPC are inactivated or removed [10,11]. Some studies have found that SPC could successfully replace part of FM in the feed of aquatic animals, such as rice field eel (*Monopterus albus*) [12], golden crucian carp (*Cyprinus carpio* × *Carassius auratus*) [13], Coho Salmon (*Oncorhynchus kisutch*) [14], golden pompano (*Trachinotus ovatus*) [15], Atlantic salmon (*Salmo salar* L.) [16], Totoaba (*Totoaba macdonaldi*) [17], red drum (*Sciaenops ocellatus*) [18], and Florida pompano (*Trachinotus carolinus*) [19], suggesting the potential of SPC to replace FM in fish feed. However, excessive replacement of FM with SPC inhibited the growth, feed utilization, and feeding of the fish to even risk its survival rate [20,21,22]. The underlying mechanism could be plant-based protein-mediated inhibition of TOR, signaling transduction and downregulation of lipolysis-related factors, thereby inducing liver metabolic disorders and inhibition of metabolism [23,24]. Hence, it is important to determine the replacement proportion of FM with SPC in aquatic animal feed.

The healthy properties of soybeans are attributed to the soy isoflavones [25,26] and their metabolites which are capable of exerting anti-inflammatory effects [27]. Bitzer et al. [28] reported that SPC had a cellular protective role in vitro, alleviating the severity of inflammation and loss of intestinal barrier function in vivo. However, plant protein sources widely used in feed could induce intestinal inflammation in carnivorous fish [29,30]. The replacement of 20% FM with SPC did not affect the intestinal health of the fish; however, replacement above 40% negatively impacted the intestinal morphology-related indicators such as microvilli length, causing obvious symptoms of enteritis in pearl gentian groupers (*Epinephelus fuscoguttatus* ♀ × *Epinephelus lanceolatus* ♂) [31]. Furthermore, the partial replacement of FM with SPC was beneficial for improving the serum antioxidant capacity in rice field eels [12]. Zhu et al. [13] demonstrated that when SPC replaced 40% FM, antioxidant enzyme activities and malondialdehyde (MDA) content were not significantly affected in golden crucian carp (*Cyprinus carpio* × *Carassius auratus*); however, a high proportion of SPC replacing FM could lead to decreased enzymes activities of glutathione peroxidase (GSH-Px) and catalase (CAT) in hybrid grouper (*Epinephelus fuscoguttatus* ♀ × *Epinephelus lanceolatus* ♂) [32] and decreased enzymes activities of GSH-Px and total superoxide dismutase in starry flounder (*Platichthys stellatus*) [20].

Largemouth bass (*Micropterus salmoides*) is a native of North America. Due to its rapid growth rate, wide temperature tolerance, versatility in adapting to different conditions, and attractiveness as a food source, it has been widely cultivated in China [33] and other parts of the world [34]. As a carnivorous fish species, largemouth bass has a high demand for FM, constituting about 40–55% of dry matter [35,36]. SPC has immense potential in replacing FM and has been applied to other aquatic animals with good results [12,13,14,15,16,17,18,19]; however, research information on largemouth bass is insufficient. Hence, this experiment was designed to study the effects of the replacement of FM with SPC on the growth, nutritional metabolism, antioxidant status, and inflammatory factors of juvenile largemouth bass.

## 2. Materials and Methods

### 2.1. Diets

A total of five feed groups were designed as SPC0, SPC25, SPC50, SPC75, and SPC100 replacing 0%, 25%, 50%, 75%, and 100% of FM with SPC in the feed, respectively (Table 1). The main protein sources, including FM, SPC, soybean meal, and corn protein meal, were sieved through a screen size of 0.18 mm before meal preparation with sequential mixing of all the raw materials according to our previously reported method [37]. 

### 2.2. Culture Experiment

Largemouth bass was procured from the Freshwater Fisheries Research Center (FFRC) followed by the culture experiment at the FFRC base. The fish were acclimatized for two weeks before the breeding experiment. After 24 h of fasting, 300 fish (17.03 ± 0.01 g) were divided into 15 cages with 20 fish per cage by the randomness principle. They were fed twice a day (at 6:30 and 18:30) until they no longer surfaced to feed. The water quality conditions were water temperature of 24–29 °C with dissolved oxygen ≥6 mg/L (pH 7.4–8.0), respectively.

### 2.3. Sample Collection

At day 56 of the breeding experiment, fish were fasted for 24 h followed by counting and weighing to determine the growth parameters. Three fish were randomly selected in each cage and anesthetized (100 mg/L MS-222) to collect the liver and intestine samples, which were stored separately in cryopreservation tubes and immediately placed in liquid nitrogen for freezing. Thereafter, the samples were kept in the refrigerator at −80 °C for further use. In addition, the intestine tissues were fixed with 4% paraformaldehyde for hematoxylin and eosin (HE) staining analysis.

### 2.4. Experimental Determination Method

The experimental feed composition and whole fish components were measured as described by AOAC [38] and our previous study [39]. Briefly, the sample was dried in an oven at 105 °C to a constant weight for testing the moisture level, and the dried sample was ground into a powder for further analysis. The crude protein was quantified by the Kjeldahl nitrogen determination method on an automatic instrument (Haineng K1100, Jinan Haineng Instrument Co., Ltd., Jinan, China). The crude lipid in the sample was extracted by the Soxhlet extraction method in an automatic fat analyzer (Haineng SOX606, Jinan Haineng Instrument Co., Ltd., China). The ash content was analyzed at 550 °C for 5 h by burning in a Muffle furnace (XL-2A, Hangzhou Zhuochi Instrument Co., Ltd., Hangzhou, China). Additionally, energy in the feed was measured using an oxygen bomb calorimeter (IKA C6000, Stauffen, Germany). The activities of intestinal antioxidant enzymes and the levels of MDA and glutathione (GSH) were detected using a kit following our previous method [40]. In brief, the activities of superoxide dismutase (SOD), CAT, and GSH-Px were tested by hydroxylamine method, ammonium molybdenum acid method, and colorimetric method, respectively. The levels of GSH and MDA were tested by microplate method and thiobarbituric acid method, respectively. Assay kits purchased from Jian Cheng Bioengineering Institute (Nanjing, China).

The intestinal tissue samples were fixed in 4% paraformaldehyde for more than 48 h. After dehydration in the alcohol gradient, samples were embedded in wax, followed by sectioning and cooling at −20 °C in a refrigerator. The frozen sections were brought to room temperature, and the section of the intestine was performed through the following steps: fixation by 4% paraformaldehyde, dehydration by gradient alcohol and methyl salicylate clearing, paraffin embedding, slicing, staining, etc. Finally, pathological changes in intestine were analyzed with a Zeiss microscope (Axioplan 2, Oberkochen, Germany). The specific method can be found in our previous study [41].

Next, RNA was extracted from the liver and intestine samples by RNAiso Plus (Vazyme, Nanjing, China) reagent. The A_260/280_ value of 1.8–2.0 served as a standard for further experiments by using the NanoDrop 2000 spectrophotometer. The primers were synthesized by Shengong Bioengineering Co., LTD (Shanghai, China). CFX96 Touch (Bio-Rad, Singapore) was used for quantitative real-time PCR detection. The *β-actin* gene was selected as the reference gene to calculate mRNA levels using the standard curve method [42] and the gene expression levels were further standardized. The primers for gene amplification are shown in Table 2.

### 2.5. Data Analysis

SPSS (20.0) was used for one-way ANOVA, and the method of Tukey was used to analyze the significant difference among all groups (*p* < 0.05). Results were expressed as mean ± standard error, with different superscript letters representing significant differences (*p* ˂ 0.05).

## 3. Results

### 3.1. Growth Performance

Table 3 shows the results of growth performance. Various growth parameters including FW, WGR, and SGR were not significantly different among SPC0, SPC25, and SPC50 groups (*p* > 0.05); however, they were significantly higher than those in SPC75 and SPC100 groups (*p* < 0.05). Conversely, significantly lower FCR was determined for the SPC0, SPC25, and SPC50 groups compared with that for the SPC100 group (*p* < 0.05); specifically, no significant difference among SPC0, SPC25, and SPC50 groups was found (*p* > 0.05). Moreover, compared with SPC75 and SPC100 groups, a significantly higher FI and SR was observed in the SPC0 group (*p* < 0.05).

### 3.2. Whole Fish Composition

The results of whole fish composition revealed no significant differences in the whole fish composition in the groups (*p* > 0.05; Table 4).

### 3.3. The mRNA Expression of Protein Metabolism-Related Genes in the Liver

**Figure 1:**
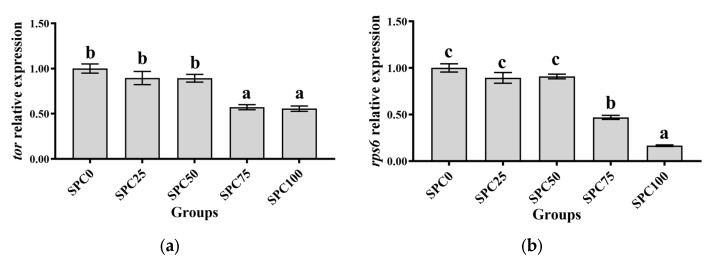

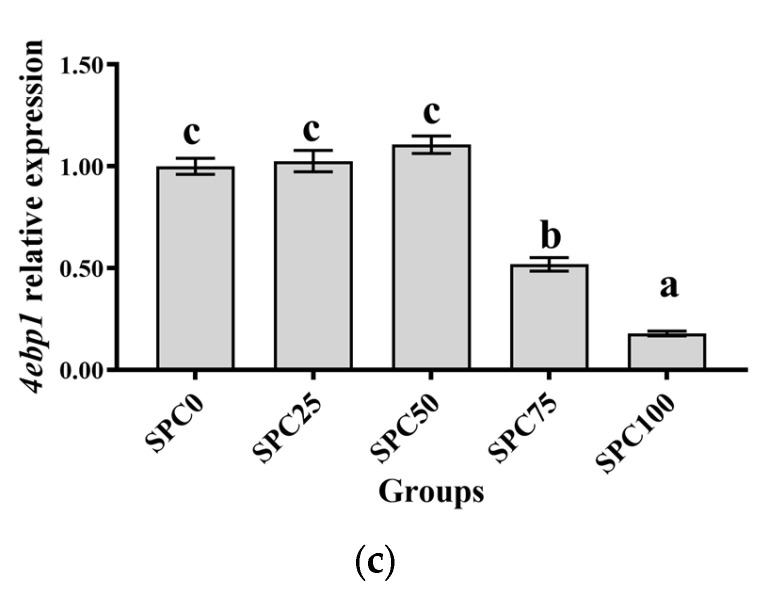
The mRNA expression of protein metabolism-related genes in the liver: (**a**) *tor*; (**b**) *rps6*; (**c**) *4ebp1*. The different letters of means are significantly different.

Results revealed no significant differences between SPC0, SPC25, and SPC50 groups in the mRNA levels of *tor*, *rps6*, and *4ebp1* (*p* > 0.05) (Figure 1a–c); however, significantly lower mRNA levels of those were found in SPC75 and SPC100 compared with the other groups (*p* < 0.05) (Figure 1a–c).

### 3.4. The mRNA Expression of Lipid and Glucose Metabolism-Related Genes in the Liver

**Figure 2:**
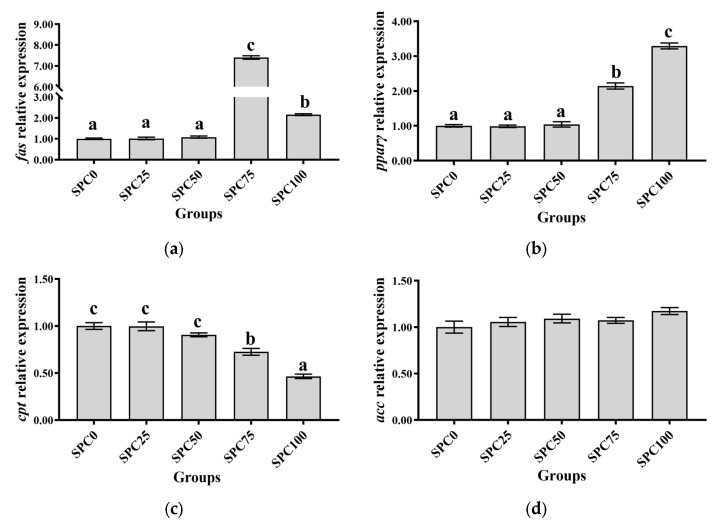

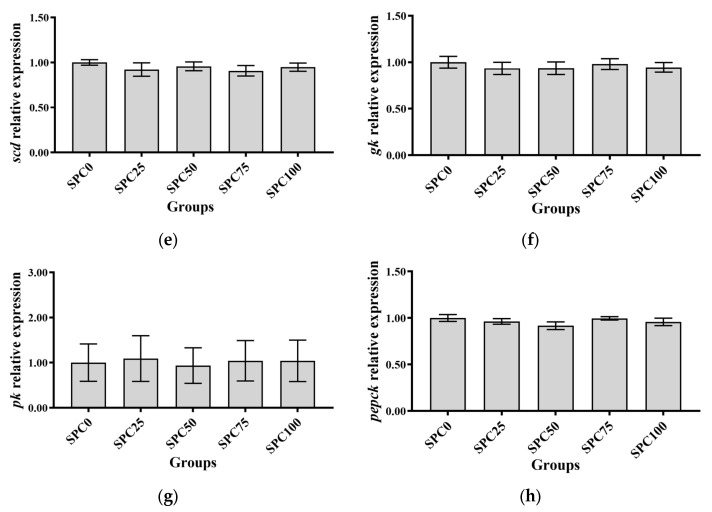
The mRNA expression of lipid and glucose metabolism-related genes in the liver: (**a**) *fas*; (**b**) *pparγ*; (**c**) *cpt*; (**d**) *acc*; (**e**) *scd*; (**f**) *gk*; (**g**) *pk*; (**h**) *pepck*. The different letters of means are significantly different.

The SPC0, SPC25, and SPC50 groups revealed no significant differences in the mRNA levels of *pparγ* and *fas* (*p* > 0.05), which were significantly lower than those in the other groups (*p* < 0.05) (Figure 2a,b). Additionally, the mRNA levels of *cpt* were significantly higher in SPC0, SPC25, and SPC50 groups than in SPC75 and SPC100 groups (*p* < 0.05) (Figure 2c). Moreover, the mRNA levels of *scd* and *acc* remained unchanged for all the groups (*p* > 0.05) (Figure 2d,e). Replacement of FM with SPC did not significantly affect the mRNA levels of *gk*, *pk*, and *pepck* (*p* > 0.05) (Figure 2f–h). 

### 3.5. Intestinal Antioxidant Parameters

**Figure 3:**
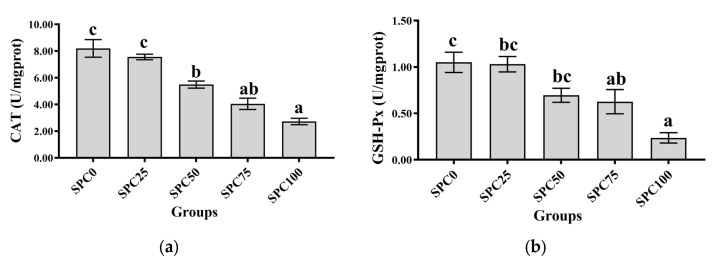

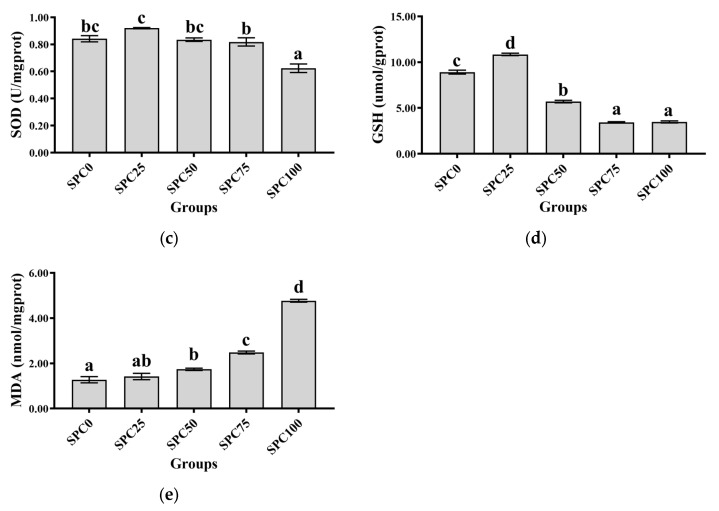
Intestinal antioxidant parameters: (**a**) CAT; (**b**) GSH-Px; (**c**) SOD; (**d**) GSH; (**e**) MDA. The different letters of means are significantly different.

Compared with the SPC0 group, significantly decreased activities of CAT were observed in the SPC50, SPC75, and SPC100 groups (*p* < 0.05) (Figure 3a), and significantly decreased activities of GSH-Px were observed in the SPC75 and SPC100 groups (*p* < 0.05) (Figure 3b). In addition, significantly lower activity of SOD was observed in SPC100 compared with the other groups (*p* < 0.05) (Figure 3c). Moreover, compared with the other groups, the SPC75 and SPC100 groups had significantly decreased and increased contents of GSH and MDA, respectively (*p* < 0.05) (Figure 3d,e). 

### 3.6. The mRNA Expression of Antioxidant Genes in the Intestine

**Figure 4:**
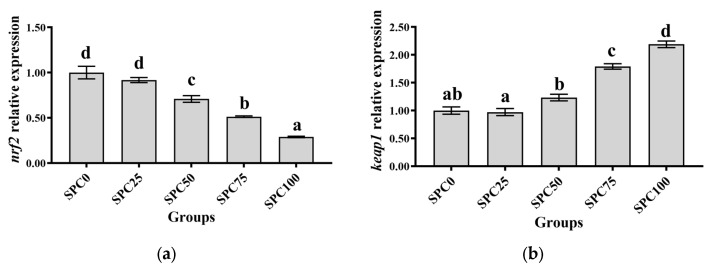

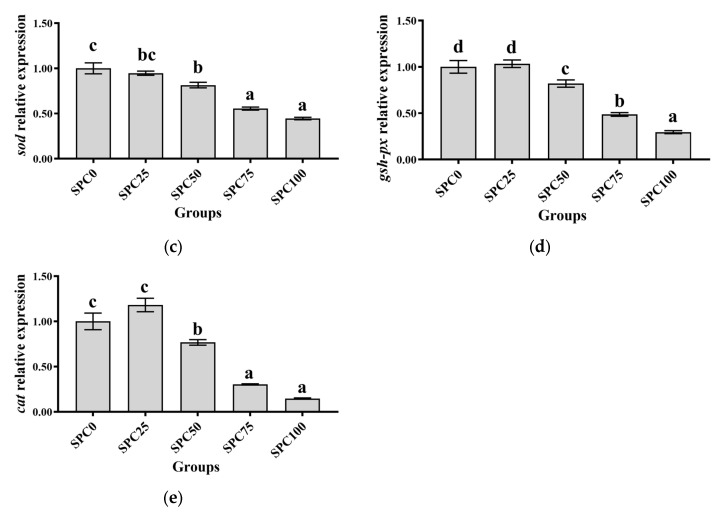
The mRNA expression of antioxidant genes in the intestine: (**a**) *nrf2*; (**b**) *keap1*; (**c**) *sod*; (**d**) *gsh-px*; (**e**) *cat*. The different letters of means are significantly different.

Compared with the SPC0 group, significantly lower mRNA levels of *nrf2*, *cat*, *sod*, and *gsh-px* were found in SPC50, SPC75, and SPC100 (*p* < 0.05) (Figure 4a,c–e); however, significantly higher mRNA levels of *keap1* were observed in SPC75 and SPC100 groups (*p* < 0.05) (Figure 4b). 

### 3.7. The mRNA Expression of Inflammatory Response-Related Genes in the Intestine

**Figure 5:**
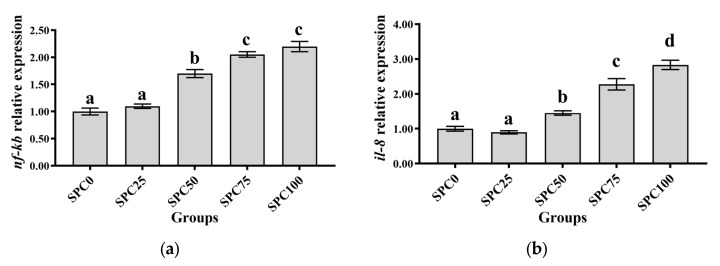

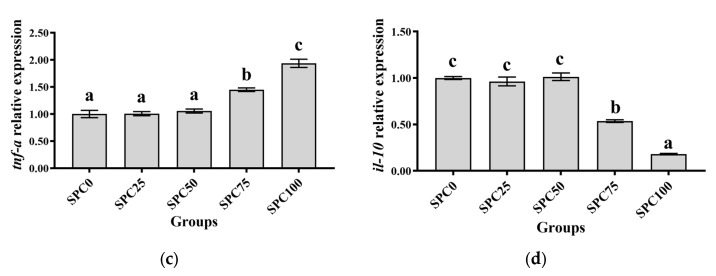
The mRNA expression of inflammatory response-related genes in the intestine: (**a**) *nf-κb*; (**b**) *il-8*; (**c**) *tnf-α*; (**d**) *il-10*. The different letters of means are significantly different.

Compared with the SPC0 group, significantly higher mRNA levels of *il-8* and *nf-κb* were found in the SPC50, SPC75, and SPC100 groups (*p* < 0.05) (Figure 5a–c). Conversely, significantly lower mRNA levels of *il-10* and significantly higher mRNA levels of *tnf-α* were found in the SPC75 and SPC100 groups compared with the other groups (*p* < 0.05) (Figure 5d). 

### 3.8. Intestinal Morphology

**Figure 6:**
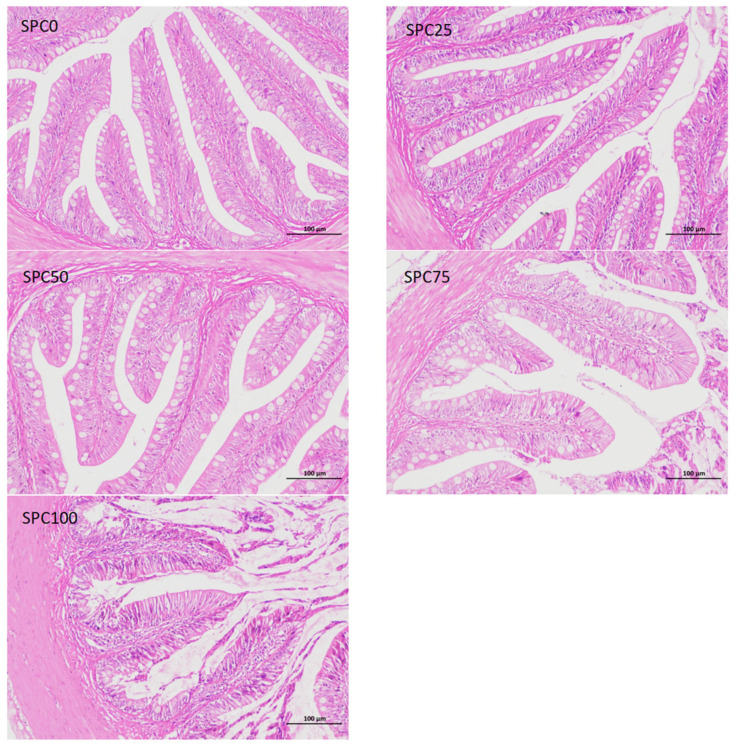

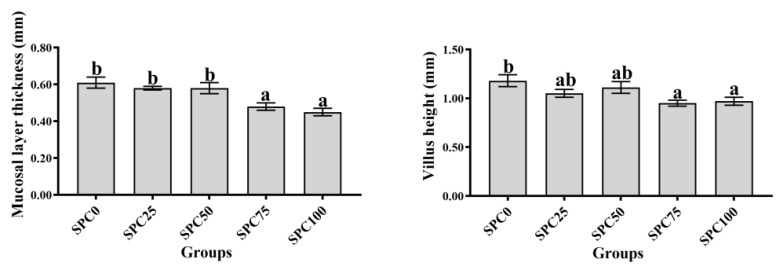
Intestinal morphology. The different letters of means are significantly different.

Compared with the SPC0 group, mucosal thickness and villus height were significantly decreased in the SPC75 and SPC100 groups (*p* < 0.05; Figure 6). 

## 4. Discussion

In the study, varying concentrations of SPC (0%, 25%, and 50%) did not affect growth-performance-related parameters, including FW, WGR, SGR, FCR, FI, and SR. This is consistent with the previous findings of a study on largemouth bass by Cui et al. [45]. Metochis et al. [46] found that replacing 35% of FM with SPC improved the growth performance of Atlantic salmon significantly, indicating the potential of SPC in replacing FM in fish feed. However, when SPC replaced 60% FM in the feed, the growth was significantly decreased, while the FCR was significantly increased [47]. Our study has shown similar results where significant inhibition of the growth and feed utilization of juvenile largemouth bass was observed when SPC replaced 75% or higher proportion of FM. Therefore, it is evident that only a specific proportion of FM should be replaced with SPC as excessive SPC negatively impacts fish growth, feed utilization, and SR. In addition, no significant difference was observed in the whole body composition between the groups, which was consistent with the previous studies of largemouth bass [45].

Protein source replacement of FM can affect the protein synthesis in largemouth bass [41,48]. Hay and Sonenberg [49] showed that the TOR signaling pathway affects protein synthesis and controls cell growth. Our results demonstrated that SPC75 and SPC100 groups inhibited the mRNA levels of *tor*, *rps6*, and *4ebp1* in the liver relative to other groups, suggesting that at 75% or higher proportion of SPC replacing FM, the expression of the TOR signaling pathway-related genes would be inhibited, thereby reducing the protein synthesis. Similar results were demonstrated in previous studies when high levels of plant protein supplementation in feed reduced mRNA levels of core genes of the TOR signaling pathway [50]. The inhibition of the TOR signaling pathway and protein synthesis could also describe the decreased growth of largemouth bass after feeding SPC75 and SPC100 diets. In addition, FM replacement with other protein sources will affect the lipid metabolism in fish [51]. Our study showed that the mRNA levels of *fas* and *pparγ* (related to lipid synthesis) were significantly upregulated in the SPC75 and SPC100 groups, while those of *cpt* (related to lipid decomposition) were significantly downregulated. Alternatively, the replacement of FM with 75% or more SPC might limit lipolysis in largemouth bass by upregulating lipid synthesis and downregulating lipolysis-related genes, thereby causing fat accumulation. In Japanese perch (*Lateolabrax japonicas*), feeding a whole plant protein diet could cause fatty liver [52]. In summary, this study revealed that a 75% or higher proportion of SPC replacing FM significantly downregulated the expression of genes related to protein anabolism and lipolysis, thereby affecting the nutritional metabolic capacity of fish and inducing liver metabolic disorders. This might be the possible reason for the significantly decreased growth performance and feed utilization of largemouth bass fed with SPC75 and SPC100 diets. Previous studies on red sea bream (*Pagrus major*) have also reported that FM-free feed with SPC resulted in poor nutrient utilization and growth inhibition in fish [53].

Furthermore, GSH-Px, CAT, and SOD are the main antioxidant enzymes in fish [54]. GSH balances redox reactions by eliminating excess ROS, thereby protecting cells from oxidative stress [55]. Our results showed that significantly decreased activities of CAT were observed in the SPC50, SPC75, and SPC100 groups, and significantly decreased activities of GSH-Px and contents of GSH were observed in the SPC75 and SPC100 groups. In addition, significantly lower activity of SOD was observed in SPC100 compared with the other groups. In addition, MDA, a lipid peroxidation product, is an important index to measure cell damage and biotoxicity [56]. Our results showed significantly increased levels of MDA in the groups of SPC75 and SPC100, which was consistent with the previous studies. SPC replacing 60% or higher proportion of FM decreased SOD activity in the serum of starry flounder, and GSH-Px activity was significantly decreased when SPC replaced 80% and 100% of FM, which also presented lower MDA level in serum [20]. Moreover, the activities of antioxidant enzymes and their related mRNA levels are correlated [57]. The Nrf2 system could regulate the expression of antioxidant genes in fish [58]. We observed that significantly lower mRNA levels of *nrf2*, *cat*, *sod*, and *gsh-px* were found in SPC50, SPC75, and SPC100 and significantly higher mRNA levels of *keap1* were observed in SPC75 and SPC100 groups. These results showed that SPC replacing 50% or more of FM could significantly decrease the intestinal antioxidant capacity of largemouth bass. In a similar study, when SPC replaced 60% of FM, the antioxidant capacity was significantly restrained in golden crucian carp [13]. In addition, our results showed that replacing 50% of FM with SPC did not affect SOD and GSH-Px activities in the intestine of largemouth bass; however, at a replacement ratio of 25%, GSH levels were significantly increased, which might be related to the presence of soybean isoflavones in SPC. Yang et al. [59] found that appropriate supplementation level of soybean isoflavones to the feed could increase the levels of GSH, SOD, and CAT in the muscle of grass carp while downregulating and upregulating the mRNA levels of *keap1* and *nrf2*, respectively. Zhou et al. [60] also found that feed supplemented with an appropriate concentration of soy isoflavones could increase the SOD and CAT activities in golden pompano.

Conversely, the decreased FM content in feed can reduce the antioxidant capacity of fish, leading to intestinal inflammation [52]. Our results demonstrated that significantly higher mRNA levels of *il-8* and *nf-κb* were found in the SPC50, SPC75, and SPC100 groups and significantly lower mRNA levels of *il-10* and significantly higher mRNA levels of *tnf-α* were found in the SPC75 and SPC100 groups. These results indicated that high levels of replacement FM through SPC might cause inflammation. Similarly, in pearl gentian groupers, replacement of 20% FM with SPC did not affect *il-10* and *tnf-α* significantly; however, significantly lower mRNA levels of anti-inflammatory factors and higher levels of proinflammatory factors were observed at 40% SPC [31]. Zhang et al. [52] found that Japanese seabass fed on whole plant protein feed caused inflammation. Chen et al. [61] also reported that 75% FM replaced with SPC in the diet of hybrid grouper caused inflammation in the fish intestines. Collectively, these findings suggested that a large proportion of SPC replacing FM might lead to an intestinal inflammatory response in largemouth bass, causing intestinal damage. Moreover, the morphology and structure of the intestine are critical for maintaining normal function and nutrient absorption [62,63,64]. Our results did not show any changes in the intestinal mucosal thickness and villus height in the SPC0, SPC25, and SPC50 groups; however, significant decreases in these parameters were found in the SPC75 and SPC100 groups relative to the SPC0 group. This might have resulted from the lack of active substances such as small peptides [65], which are abundantly present in FM and necessary for intestinal development and health; therefore, a significant reduction in FM decreased villous height in the intestines of largemouth bass. However, our results showed that reduced intestinal mucosal thickness and villi height in the SPC75 and SPC100 groups might cause malabsorption of nutrients in fish, thereby leading to slow growth of largemouth bass.

## 5. Conclusions

SPC replacing 50% FM in the feed did not affect growth. Furthermore, SPC replacing 75% or more FM could inhibit protein synthesis by lowing the gene expressions of *tor*, *rps6*, and *4ebp1*, and might limit lipolysis in largemouth bass by upregulating lipid synthesis and downregulating lipolysis-related genes including *pparγ*, *fas*, and *cpt*, while there was no effect on the expression of genes related to glucose metabolism including *gk*, *pk*, and *pepck*. However, SPC replacing 50% or more FM might inhibit antioxidant capacity and immune capacity by regulating antioxidant enzyme activity and gene expressions. Furthermore, SPC replacing 75% or more FM could reduce the thickness of intestinal mucosa and villus height.

## Figures and Tables

**Table 1:** Experimental formula (dry matter, %).

Ingredients	SPC0	SPC25	SPC50	SPC75	SPC100
Soy protein concentrate ^1^	0	11.5	23.1	34.7	46.3
Fish meal ^1^	45	33.75	22.5	11.25	0
Soybean meal ^1^	12	12	12	12	12
corn gluten meal ^1^	13	13	13	13	13
Cassava starch	5	5	5	5	5
Wheat flour	5	5	5	5	5
Rice bran	3.36	3.36	3.36	3.36	3.36
Microcrystalline cellulose	9.04	6.904	4.566	2.285	0
Fish oil	3.9	4.5	5.1	5.7	6.3
Vitamins premix ^2^	1	1	1	1	1
Mineral premix ^2^	1	1	1	1	1
Calcium dihydrogen phosphate	1.2	2.2	3.2	4.2	5.2
Choline chloride	0.5	0.5	0.5	0.5	0.5
Lysine	0	0.169	0.335	0.5	0.666
Methionine	0	0.117	0.234	0.35	0.467
Threonine	0	0	0.057	0.085	0.113
Valine	0	0	0.048	0.07	0.093
Proximate analysis (dry basis)					
Crude protein (%)	47.21	47.09	47.05	46.82	46.83
Crude lipids (%)	9.42	9.92	9.94	9.91	9.87
Gross energy (kJ/g)	17.13	16.98	17.01	17.08	16.99

Note: ^1^ The crude protein and crude lipids levels of the raw material are shown below, respectively. Fish meal, 67.63% and 9.46% g; soy protein concentrate, 63% and 4.1%; soybean meal, 53.26% and 4.25%; corn gluten meal, 59.24% and 3.3%. These materials were obtained from Wuxi Tongwei feedstuffs Co., Ltd. (Wuxi, China) ^2^ Mineral premix and vitamins premix were obtained from HANOVE Animal Health Products (Wuxi, China).

**Table 2:** Experimental primer.

Genes	Forward (5′-3′)	Reverse (5′-3′)	Primer Source
*tor* ^1^	TTTGGAACCAAACCCCGTCA	ATCAGCTCACGGCAGTATCG	XM_038723321.1
*rps6* ^2^	TCCAGAGACTCGTGACACCT	AGCTTGGCATACTCTGAGGC	XM_038713349.1
*4ebp1* ^3^	CCAGGATCATCTATGACCGAAAG	TGCAGCGATATTGTTGTTGTTC	XM_038703879.1
*fas* ^4^	AGTTGAAGGCTGCTGATG	GCTGTGGATGATGTTGGT	XP_028423094.1
*acc* ^5^	TTACATCGCAGCCAACAG	CTCTCCACCTTCCTCTACA	XP_022609673.1
*scd* ^6^	CGATGCTGCTTCTTCACT	GACACGGTTCTGCCATTA	XM_038735580.1
*cpt* ^7^	TTACCGTATGGCTATGACTG	GGCTCCGATAACACCTCT	XP_027141042.1
*pparγ* ^8^	GAGTTCTCAGTCAAGTTCAAC	AATGTAGCACCGTCTCCT	MK614721.1
*gk* ^9^	CCCTTGTGGGCAGGAGAAAA	ACAACTGAGTCCTCCTTGCG	XP_023260296.1
*pk* ^10^	CACGCAACACTGGCATCATC	TCGAAGCTCTCACATGCCTC	MT431526.1
*pepck* ^11^	GGCAAAACCTGGAAGCAAGG	ATAATGGCGTCGATGGGGAC	MT431525.1
*nrf2* ^12^	CCACACGTGACTCTGATTTCTC	TCCTCCATGACCTTGAAGCAT	Transcriptome data
*cat* ^13^	CTATGGCTCTCACACCTTC	TCCTCTACTGGCAGATTCT	MK614708.1
*sod* ^14^	CCCCACAACAAGAATCATGC	TCTCAGCCTTCTCGTGGA	MK614709.1
*gsh-px* ^15^	ATGGCTCTCATGACTGATCCAAA	GACCAACCAGGAACTTCTCAAA	MK614713.1
*keap1* ^16^	GCACCTAACCGTGGAACTCAA	CCAGTTTTAGCCAGTCATTGTTCC	[43]
*nf-κb* ^17^	AGAAGACGACTCGGGGATGA	GCTTCTGCAGGTTCTGGTCT	[44]
*il-8* ^18^	GAGGGTACATGTCTGGGGGA	CCTTGAAGGTTTGTTCTTCATCGT	XM_038713529.1
*tnf-α* ^19^	CTTCGTCTACAGCCAGGCATCG	TTTGGCACACCGACCTCACC	[36]
*il-10* ^20^	CGGCACAGAAATCCCAGAGC	CAGCAGGCTCACAAAATAAACATCT	[36]
*β-actin*	ATGCAGAAGGAGATCACAGCCT	AGTATTTACGCTCAGGTGGGG	AF253319.1

Note: ^1^ *tor*, target proteins rapamycin; ^2^ *rps6*, ribosomal protein S6 kinase; ^3^ *4ebp1*, eukaryotic initiation factor 4E-binding protein 1; ^4^ *fas*, fatty acid synthetase; ^5^ *acc*, acetyl-CoA carboxylase; ^6^ *scd*, stearoyl-CoA desaturase; ^7^ *cpt*, carnitine palmitoyl transferase; ^8^ *pparγ*, peroxisome proliferator-activated receptor-γ; ^9^ *gk*, glucokinase; ^10^ *pk*, pyruvate kinase; ^11^ *pepck*, phosphoenolpyruvate carboxylase; ^12^ *nrf2*, nuclear factor E2 related factor 2; ^13^ *cat*, catalase; ^14^ sod, superoxide dismutase; ^15^ *gsh-px*, glutathione peroxidase; ^16^ *keap1*, Kelch-like ECH associated protein 1; ^17^ *nf-κb*, nuclear factor kappa B; ^18^ *il-8*, interleukin-8; ^19^ *tnf-α* tumor necrosis factor α; ^20^ *il-10*, interleukin-10.

**Table 3:** Growth performance.

Groups	IW (g) ^1^	FW (g) ^2^	WGR (%) ^3^	SGR (%/d) ^4^	FCR ^5^	FI (g fish^−1^d^−1^) ^6^	SR (%) ^7^
SPC0	17.07 ± 0.02	58.38 ± 0.13 ^c^	242.08 ± 0.91 ^c^	2.20 ± 0.00 ^c^	1.40 ± 0.03 ^a^	0.52 ± 0.00 ^c^	96.67 ± 1.67 ^c^
SPC25	17.03 ± 0.02	57.96 ± 0.75 ^c^	240.25 ± 4.17 ^c^	2.19 ± 0.02 ^c^	1.41 ± 0.04 ^a^	0.51 ± 0.01 ^bc^	95.00 ± 2.89 ^bc^
SPC50	17.02 ± 0.02	57.18 ± 1.95 ^c^	236.06 ± 11.71 ^c^	2.16 ± 0.06 ^c^	1.54 ± 0.04 ^a^	0.50 ± 0.01 ^bc^	88.33 ± 1.67 ^abc^
SPC75	17.03 ± 0.02	51.07 ± 0.63 ^b^	199.84 ± 3.66 ^b^	1.96 ± 0.02 ^b^	1.76 ± 0.12 ^ab^	0.48 ± 0.00 ^ab^	85.00 ± 2.89 ^ab^
SPC100	17.02 ± 0.02	40.78 ± 1.31 ^a^	139.65 ± 7.76 ^a^	1.56 ± 0.06 ^a^	1.99 ± 0.13 ^b^	0.45 ± 0.01 ^a^	83.33 ± 1.67 ^a^

Note: All data are expressed as mean ± standard error. Means in the same column with different superscripts are significantly different (*p* < 0.05). ^1^ IW, initial average weight. ^2^ FW, final average weight. ^3^ Weight gain rate (WGR, %) = 100 × (final body weight (g) − initial body weight (g))/initial body weight (g). ^4^ Specific growth rate (SGR, %/d) = 100 × ((Ln (final body weight (g)) − Ln (initial body weight (g)))/days). ^5^ Feed conversion ratio (FCR) = dry feed fed (g)/wet weight gain (g). ^6^ Feed intake rate (FI, g fish^−1^d^−1^) = dry feed fed (g)/((total initial weight (g) + total final weight (g))/2/days). ^7^ Survival rate (SR, %) = 100 × (survival fish number/total fish number).

**Table 4:** Whole fish composition.

Groups	Moisture (%)	Ash (%)	Lipids (%)	Protein (%)
SPC0	71.72 ± 0.33	3.49 ± 0.12	6.94 ± 0.23	16.59 ± 0.35
SPC25	71.08 ± 0.32	4.18 ± 0.31	6.45 ± 0.32	16.59 ± 0.37
SPC50	71.64 ± 0.21	4.42 ± 0.36	6.47 ± 0.78	16.92 ± 2.22
SPC75	71.81 ± 0.29	4.14 ± 0.42	5.69 ± 0.22	16.43 ± 2.11
SPC100	72.42 ± 0.29	4.18 ± 0.30	5.05 ± 0.21	16.27 ± 1.62

Note: All data are expressed as mean ± standard error.

## Data Availability

Data are contained within the article.
